# RNAi mediated inhibition of viroid infection in transgenic plants expressing viroid-specific small RNAs derived from various functional domains

**DOI:** 10.1038/srep17949

**Published:** 2015-12-14

**Authors:** Charith Raj Adkar-Purushothama, Atsushi Kasai, Kohei Sugawara, Hideki Yamamoto, Yuto Yamazaki, Ying-Hong He, Nobuyuki Takada, Hideki Goto, Sahori Shindo, Takeo Harada, Teruo Sano

**Affiliations:** 1 Faculty of Agriculture and Life Science, Hirosaki University, Bunkyo-cho 3, Hirosaki 036-8561, Japan

## Abstract

Previous attempts to develop RNAi-mediated viroid-resistant transgenic plants using nearly full-length *Potato spindle tuber viroid* (PSTVd) hairpin RNA (hpRNA) were successful; however unusual phenotypes resembling viroid infection occurred. Therefore, in the present work, transgenic *Nicotiana benthamiana* lines expressing both partial and truncated versions of PSTVd hpRNA were developed. Specifically, seven partial or truncated versions of PSTVd sequences were selected according to the hotspots of both PSTVd-sRNAs and functional domains of the PSTVd. A total of 21 transgenic lines *Nicotiana benthamiana* were developed under the control of either the CaMV-35S or the CoYMV promoters. All of the transgenic lines established here were monitored for the induction of phenotypic changes, for PSTVd-sRNA expression and for the resistance against PSTVd infection. Additionally, this study demonstrates the use of inverted repeat construct sequences as short as 26- to -49 nucleotides for both the efficient expression of the PSTVd-sRNA and the inhibition of PSTVd infection.

The term ‘viroid’ was coined in the early 1970s to describe a novel class of naked RNA pathogens similar to, but different from conventional viruses. Viroids are highly structured, non-coding, single-stranded, circular RNA molecules of 246- to 401-nucleotides (nts) in length^1^. In susceptible host plants, viroid infection often induces a wide array of symptoms, such as stunting, leaf distortion and necrosis^2^. Viroids are broadly divided into two families: *Pospiviroidae* and *Avsunviroidae*. Members of the *Pospiviroidae* family replicate in the nucleus via an asymmetrical rolling-circle mechanism and contain five structural/functional domains: the terminal left (TL), the pathogenicity (P), the central conserved region (CCR), the variable (V) and the terminal right (TR) domains. Conversely, the members of *Avsunviroidae* family replicate in the chloroplast via a symmetrical rolling-circle mechanism and exhibit autocatalytic self-cleavage activities^3^,^4^.

With the globalisation of agriculture, viroids have become widely distributed into both new environments and new geographical areas. *Potato spindle tuber viroid* (PSTVd), a representative member of *Pospiviroidae* family, is known to infect economically important plants such as *Solanum tuberosum* (potato) and *S. lycopersicum* (tomato). Recently, an asymptomatic association of PSTVd has been reported with many ornamental plants, such as *S. jasminoides*, *S. rantonnetii, Brugmansia sp*. and *Dahlia* sp., and globally has become a major threat to plant quarantine^5^. The transmission of PSTVd to susceptible crop plants, such as potato and tomato, is a potential threat to global food safety. Consequently, it is essential to develop reliable control measures in order to protect crops from these pathogens.

Due to their high degree of internal base pairing and their RNA-RNA mode of replication, viroids are strong inducers of RNA silencing and; therefore, infected host plants are often associated with the accumulation of viroid-specific small RNAs (or viroid-derived sRNAs; vd-sRNAs) 21- to 24-nts in length^6^,^7^,^8^,^9^,^10^,^11^. These vd-sRNAs are unable to completely inhibit viroid replication because viroids are somewhat tolerant to RNA silencing mechanisms and can continue to replicate in infected plants even after the induction of RNA silencing^12^. However, an RNA silencing-based strategy presents a very attractive method to create viroid-resistant (or tolerant) plants, as PSTVd-infected tomato plants exhibited ‘recovery’ in the latter stage of infection after the induction of RNA silencing^13^. Transgenic tomato plants expressing PSTVd-specific small RNAs (sRNA) showed increased resistance to PSTVd infection, whereas the co-inoculation of artificial viroid double-stranded RNA (dsRNA) inhibited homologous viroid replication in plants^14^,^15^.

The introduction and expression of an inverted repeat (IR) sequence homologous to a part of the targeted viral genome is an efficient method by which to induce the host RNA silencing machinery and thus confer viral resistance to plants^16^. Generally, a complete or nearly full-length virus gene is used to construct IR hairpins^17^. However, transgenic tomato plants created with nearly full-length PSTVd IR hairpin constructs exhibited viroid-associated symptoms^18^, suggesting that the transgenic expression of nearly full-length hairpin viroid-RNA in plants could potentially cause adverse side effects during plant growth via RNA silencing by knocking down/blocking the expression of certain endogenous genes. In addition, the transgenic expression of artificial microRNA corresponding to sequences located within the PSTVd virulence-modulating region (VMR) induced abnormal phenotypes in *Nicotiana* species that closely resembled those displayed by PSTVd-infected plants^19^. Therefore, in order to develop an efficient RNAi strategy to protect plants from viroid infection, it is essential to identify the region that triggers the viroid-targeting RNA silencing without causing adverse side effects to the host plant’s metabolism. The profiling of the high-throughput sequencing data of vd-sRNA accumulated in PSTVd-infected tomato plants revealed the presence of vd-sRNA hotspot regions on both the PSTVd genomic (plus) and antigenomic (minus) strands^20^, indicating that these regions of the viroid are more susceptible to the host’s RNA silencing mechanism. These findings prompted the creation of transgenic plants expressing hpRNAs derived from either partial or individual structural/functional domains of PSTVd in order to examine their abilities to resist/inhibit PSTVd infection/replication and to induce abnormal phenotypes. In this manuscript, a series of transgenic *Nicotiana benthamiana* (*N. benthamiana*) lines were produced using various PSTVd structural/functional domains and vd-sRNA hotspots to induce the RNAi-mediated resistance against viroid infection. The potential implications of this work are discussed with regard to RNA silencing-based strategies against viroid infection.

## Results

### Evaluation of PSTVd-specific sRNA hotspots

**Figure 1:**
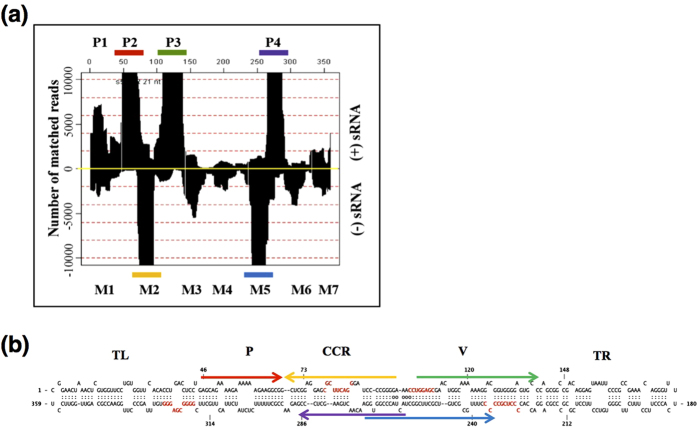
Profiling of PSTVd-sRNA for the determination of the sRNA producing hotspots. **(a)** During replication, PSTVd forms an RNA/RNA duplex which is believed to induce RNA silencing. The high-throughput sequence data obtained from tomato plants infected with PSTVd variants was mapped against the full-length PSTVd genome (1 to 359 from left to right). The upper panel indicates accumulated PSTVd-sRNA reads derived from the genomic (+) strand, and the lower panel indicates those from the antigenomic (−) strand. The profile revealed the presence of at least four PSTVd-sRNA producing hotspots on (+) strand, P1, P2, P3 and P4, and at least seven hotspots on (−) strand, M1, M2, M3, M4, M5, M6 and M7. Out of these, five regions were considered as ‘major hotspots’ as they tend to produce at least 20% of the total PSTVd-sRNA. The colored bars show these major hotspots. **(b)** Major hotspot regions were plotted on the predicted secondary structure of PSTVd. The arrow indicates the direction of the PSTVd-sRNA on the PSTVd genome. The arrows in red, green and purple represent PSTVd-sRNA hotspot regions derived from the genomic (+) strand, while the arrows in yellow and blue denote those derived from the antigenomic (−) strand.

In order to predict the regions of the PSTVd genome that are most susceptible to viroid-induced RNA silencing, the profiles of PSTVd-specific sRNA (PSTVd-sRNA) accumulation were re-examined based on the high-throughput sequence data obtained in previous experiments using PSTVd isolates and susceptible tomato cultivars^2^,^11^,^20^,^21^. PSTVd-sRNA accumulation patterns somewhat differed depending on the analytical method used, although similar hotspots were found irrespective of the host cultivar, viroid strain, growing conditions or season. Detailed analysis of these data showed that PSTVd-sRNA are 21, 22, 23 and 24-nt (a large majority were 21- and 22-nt) in length, and that certain regions of the PSTVd genome (i.e. hotspots) were more susceptible to RNA silencing than others and produced greater amounts of PSTVd-sRNA with the polarities of both the genomic or antigenomic strands (For more details see Wang *et al.*^20^ and Tsushima *et al.*^11^). The cumulative profiling of these PSTVd-sRNAs underscored the presence of at least four and seven hotspots on the genomic and antigenomic strands of the PSTVd, respectively (Fig. 1a). For convenience, the regions producing more than 20% of the total PSTVd-sRNA reads were considered major hotspots (P2 [nucleotides 47 to 70 of the PSTVd genomic strand], P3 [nucleotides 105 to 140 of the PSTVd genomic strand], P4 [nucleotides 256 to 283 of the PSTVd genomic strand], M2 [nucleotides 71 to 100 in the PSTVd antigenomic strand] and M5 [nucleotides 232 to 267 of the PSTVd antigenomic strand]), whereas those producing in between 10 and 20% of the PSTVd-sRNA were considered as being minor hotspots (P1 [nucleotides 1 to 30 of the PSTVd genomic strand], M1 [nucleotides 10 to 35 of the PSTVd antigenomic strand], M3 [nucleotides 140 to 165 of the PSTVd antigenomic strand], M4 [nucleotides 180 to 210 of the PSTVd antigenomic strand], M6 [nucleotides 290 to 315 of the PSTVd antigenomic strand] and M7 [nucleotides 330 to 359 of the PSTVd antigenomic strand]). Merging of the PSTVd-sRNA profiles *in silico* in order to predict PSTVd secondary structures revealed that each of the major hotspots was located in either the P, the CCR or the V domains (Fig. 1b). Surprisingly, the CCR domain accounted for the highest number of hotspots, indicating that this PSTVd domain is highly susceptible, or is more prone to, RNA silencing.

### Selection of PSTVd regions for IR constructs

**Figure 2:**
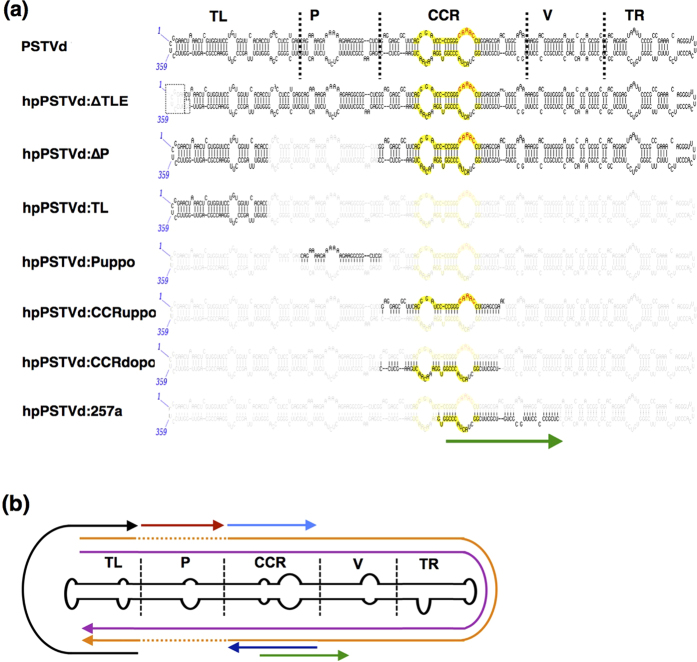
Regions of PSTVd used to produce the inverted repeat constructs. **(a)** PSTVd consists of five structural/functional domains. These are shown on the secondary structure of PSTVd, which was used for constructing various hairpin PSTVd (hpPSTVd). The yellow coloured region represents the CCR. The visible sequences are the regions that were used to make the constructs hpPSTVd:ΔTLE, hpPSTVd:TL, hpPSTVd:ΔP, hpPSTVd:Puppo, hpPSTVd:CCRuppo, hpPSTVd:CCRdopo and hpPSTVd-:257a. TL; terminal left, P; pathogenicity, CCR; conserved central region, V; variable region, TR; terminal right. The green coloured arrow indicates the anti-genomic direction used for hpPSTVd:257a construct. **(b**) Schematic view of all of the PSTVd regions used to produce inverted repeat constructs. The arrows indicate the PSTVd sequences used.

In order to produce transgenic *N. benthamiana* plants expressing various PSTVd sequence-based hpRNAs, PSTVd regions were selected by considering the effect of both the structural/functional domains and the PSTVd-sRNA producing regions (Fig. 2a,b). A total of seven transgene constructs with IR sequences were selected: (i) hpPSTVd:ΔTLE, a truncated IR construct that is a nearly full-length PSTVd-intermediate (PSTVd-I) strain derived sequence that lacks the terminal left end nucleotides (nt) (nucleotides 356 to 15) that are present in the genome^22^; (ii) hpPSTVd:ΔP, an IR construct that was created by deleting both the upper and the lower strands of the P domain from the PSTVd genome; (iii) hpPSTVd:TL, an IR construct that was created from a 91-nt sequence derived from the TL domain of the PSTVd genome; (iv) hpPSTVd:Puppo, an IR construct that was created from a 26-nt sequence of the upper strand of the P domain of the PSTVd genome; (v) hpPSTVd:CCRuppo, an IR construct that was created from a 49-nt sequence from the upper strand of the CCR domain of the PSTVd genome; (vi) hpPSTVd:CCRdopo, an IR construct that was created from a 46-nt sequence from the lower strand of the CCR domain of the PSTVd genome; and, (vii) hpPSTVd:257a, an IR construct that was created from a 40-nt sequence that corresponded to the lower ‘CCR’ and ‘V’ domains of the PSTVd-sRNA of the antigenomic strand of PSTVd (nucleotides 229 to 268). This region was specifically selected because it is located in the stem of secondary hairpin II, an alternate structure formed by base-pairing between nucleotides 227 to 236 and nucleotides 319 to 328^23^. Each of these transgene constructs was designed to correspond to at least one of the hotspot regions of the PSTVd-sRNAs, although hpPSTVd:TL contains only minor hotspots P1 and M1 (Table 1).

### Selection of *N. benthamiana* lines expressing hairpin PSTVd RNAs

**Figure 3:**
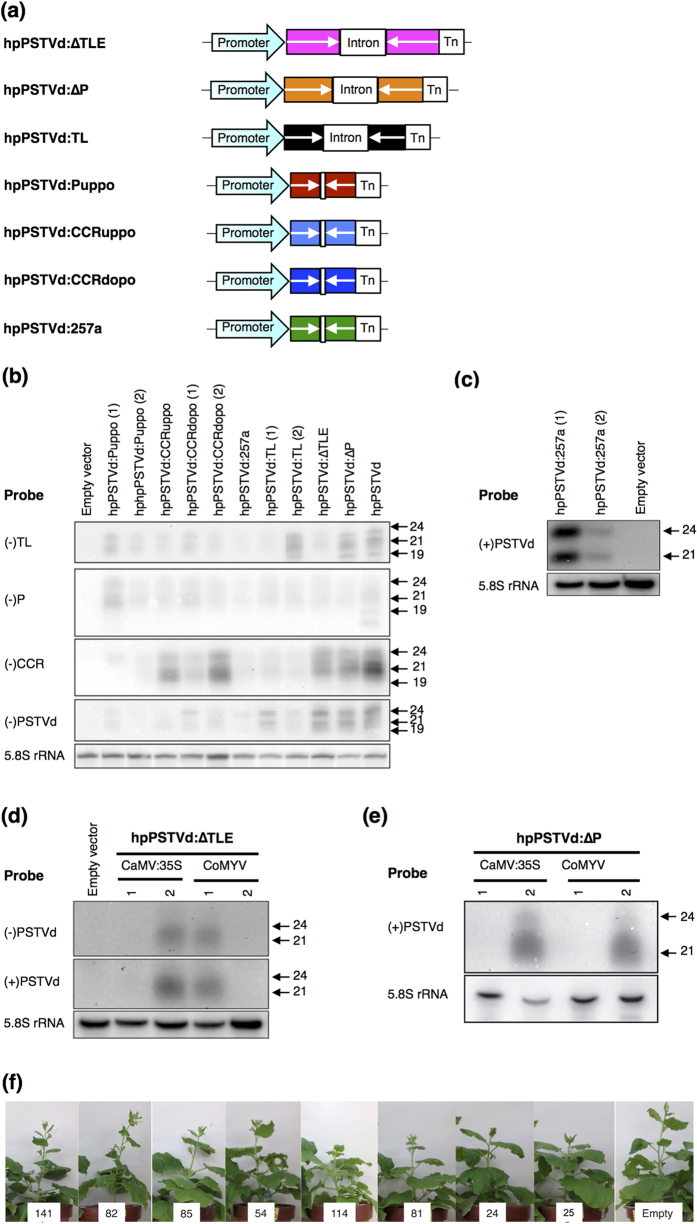
Selection of the transgenic *N. benthamiana* lines. (**a**) Schematic diagrams of all of the hpPSTVd constructs. The arrows indicate the PSTVd sequences used. The head-to-head sequences in the hpPSTVd:∆TLE, hpPSTVd:∆P and hpPSTVd:TL were separated by intron sequences whereas a small spacer sequence was used in all other constructs. In order to evaluate the production of PSTVd-sRNA by each of the IR constructs under the control of CaMV-35S promoter, *N. benthamiana* leaves were agroinfiltrated with the IR constructs and total RNA was extracted at 3-dpi and was subjected to an RNA gel blot hybridisation assay using DIG-labelled (**b**) (−) TL (antisense-TL), (−) P (antisense-P), (−) CCR (antisense-TCC), (−) PSTVd (antisense-PSTVd) and (**c**) (+) PSTVd (sense-PSTVd)-riboprobe. The amount of siRNA produced by **(d)** hpPSTVd:∆TLE and **(e)** hpPSTVd:∆P under the control of either CaMV-35S and CoMYV promoters were verified by RNA gel blot assay. Lane 1 and 2 represents the different clones of the IR constructs. **(f)** T3 generations of the selected transgenic *N. benthamiana* lines containing hp-PSTVd constructs. From left to right, 141 (hpPSTVd:∆TLE-141), 82 (hpPSTVd:∆P-82), 85 (hpPSTVd:TL-85), 54 (hpPSTVd:Puppo-54), 114 (hpPSTVd:CCRuppo-114), 81 (hpPSTVd:CCRdopo-81), 24 (hpPSTVd:257a-24), 25 (hpPSTVd:257a-25), and Empty (Empty vector).

In order to obtain the IR constructs, double-stranded cDNA of all of the selected sequences of PSTVd-I were ligated to either side of the intron or spacer sequences in a head-to-head orientation (Fig. 3a). The resulting obtained constructs were then ligated into pIG121 under the control of either the *cauliflower mosaic virus* 35S (CaMV-35S) promoter or the phloem companion cell specific *commelina yellow mottle virus* (CoMYV) promoter^22^,^24^. The resulting binary vector was then transformed into *Agrobacterium tumefaciens* (*A. tumefaciens*) and agroinfiltrated into *N. benthamiana* leaves for the transient expression of PSTVd-sRNA. At 3 days post inoculation (dpi), total RNA was extracted from the agroinfiltrated leaves and subjected to RNA gel blot assay using Digoxigenin (DIG)-labelled either partial or full length PSTVd cRNA probes in order to verify the production of PSTVd-sRNA by each of the IR constructs. As shown in Fig. 3b,c, all of the tested IR constructs produced PSTVd-sRNA, indicating that the transcribed IR products were processed into sRNAs by Dicer. Furthermore, the siRNA produced by the IR constructs under the control of both CaMV-35S and CoMYV were analysed by RNA gel blot assay and only the IR constructs capable of producing equal amount of siRNAs with both the promoters were used for the production of transgenic plants (Fig. 3d,e).

Transgenic *N. benthamiana* lines were produced by agrobacterium-mediated transformation of all of the IR constructs into *N. benthamina* as described previously^22^. These transgenic plants were expected to express hpRNAs containing a partial PSTVd sequences which would trigger RNA silencing by targeting the corresponding PSTVd sequence and thus accumulate PSTVd-sRNA. After screening by kanamycin resistance (at a segregation ratio of resistance to sensitive of 3:1) and genomic PCR analysis in order to be able to detect the promoter sequence in the plant, several transgenic lines possessing a single transgene copy were selected for each of the transgene constructs (Table 1). Other than the hpPSTVd:ΔTLE and hpPSTVd:CCRdopo lines, all others under the control of both the CaMV-35S and the CoYMV promoters were successfully obtained. In the cases of the hpPSTVd:ΔTLE and hpPSTVd:CCRdopo lines, only one was obtained for each: that under the control of the CoYMV and CaMV-35S promoter, respectively (Table 1). The shortest IR stem sequence used in this study was 26-nt in length in hpPSTVd:Puppo, followed by a 40-nt sequence in hpPSTVd:257a. The longest was 340-nt in hpPSTVd:ΔTLE, which consisted of the nearly full-length sequence of the PSTVd-I genome.

All of the T0 transgenic plants were self-pollinated in order to obtain homozygous T2 and T3 plants. Two weeks after sowing T3 seeds, transgenic seedlings were transferred to rockwool and grown in a plant growth chamber. None of these transgenic plants exhibited any noticeable phenotypic abnormality (Fig. 3f). These data indicate that the PSTVd regions selected to produce the transgenic lines do not cause any adverse effects on host’s metabolism.

### Production of PSTVd-sRNA in transgenic lines

**Figure 4:**
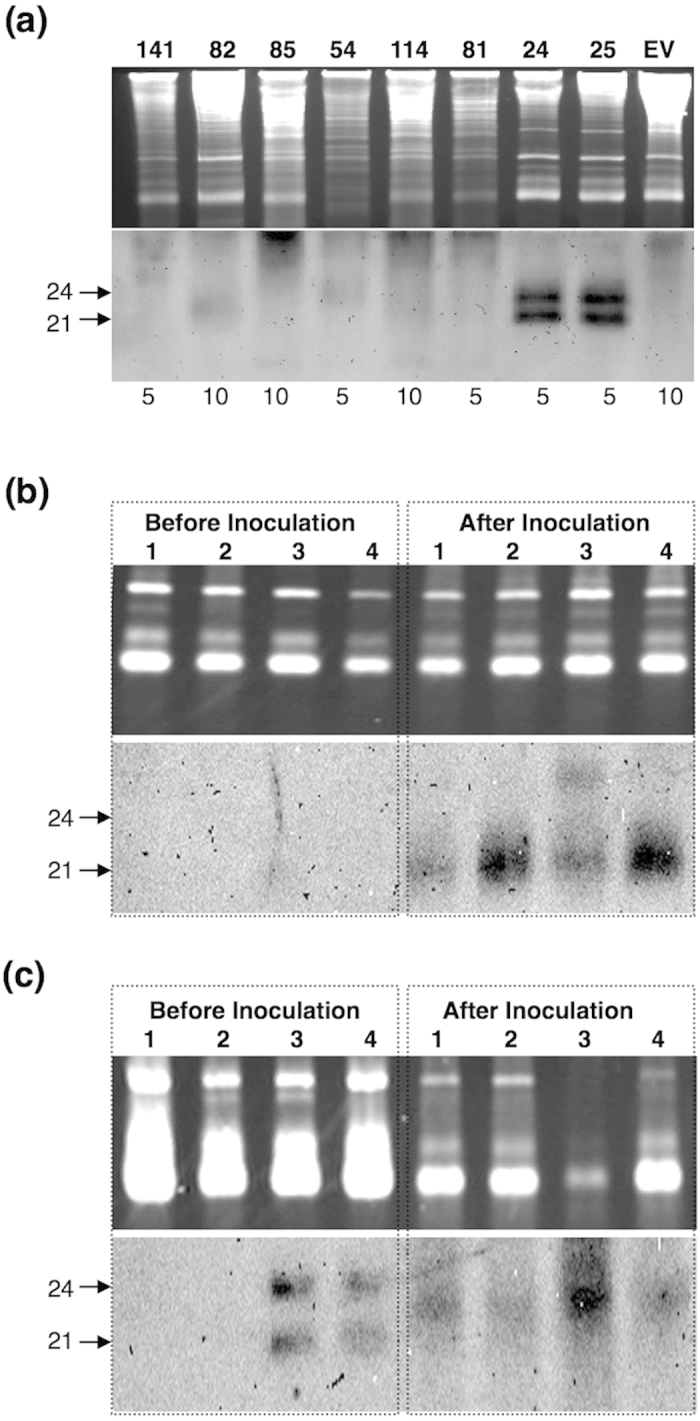
Gel blot analysis of PSTVd-sRNA production in the selected transgenic *N. benthamiana* plants. **(a)** In order to evaluate the amount of PSTVd-sRNA produced by each transgenic line, the samples were collected prior to infection assay and were analysed by gel-blot hybridisation assay using DIG-labelled PSTVd-riboprobe. The upper panel represents the ethidium bromide stained 12% PAGE containing 8M urea. *Lane 141* hpPSTVd:∆TLE-141, *Lane 82* hpPSTVd:∆P-82, *Lane 85* hpPSTVd:TL-85, *Lane 54* hpPSTVd:Puppo-54, *Lane 114* hpPSTVd:CCRuppo-114, *Lane 81* hpPSTVd:CCRdopo-81, *Lane 24* hpPSTVd:257a-24, *Lane 25* hpPSTVd:257a-25 and *Lane EV* Empty vector. The numbers below each lane represent the μg of RNA charged per lane. To demonstrate the production of sRNA by PSTVd upon infection, the samples were collected both before and after the PSTVd infection assay and were analysed by gel-blot hybridisation assay using DIG-labelled PSTVd-riboprobe after separation on 12% PAGE containing 8M urea. (**b**) *Lane 1* Empty vector, *Lane 2* hpPSTVd:∆TLE-24, *Lane 3* hpPSTVd:∆TLE-32 and *Lane 4* hpPSTVd:∆TLE-141. (**c**) *Lane 1* Empty vector, *Lane 2* hpPSTVd:257a-60, *Lane 3* hpPSTVd:257a-24 and *Lane 4* hpPSTVd:257a-25.

In order to evaluate the accumulation of PSTVd-sRNA in each transgenic *N. benthamiana* line, RNA gel blot hybridisation was performed using low molecular weight RNAs (LMW RNA) extracted from young expanded leaves of T3 seedlings (7 to 8-true leaf stage) and a DIG-labeled PSTVd cRNA probe. The amount of PSTVd-sRNA accumulated in the plant varied depending on the transgenic line, regardless of the transgene construct and promoter involved. For instance, two lines (i.e. 24 and 25) of hpPSTVd:257a under the control of the CaMV-35S promoter accumulated the highest amounts of PSTVd-sRNA (Fig. 4a), an amount comparable to that induced by native PSTVd infection (i.e. >1 μg of LMW-RNA was sufficient to detect strong PSTVd-sRNA signals by RNA gel blot hybridisation analysis). Notably, these transgenic lines exhibited the same PSTVd-sRNA pattern as two size classes of PSTVd-sRNA of 21- and 24-nt were clearly visible. This was in contrast with the three major size classes (21-, 22- and 24-nt) induced by native PSTVd infection (Figure S1). HpPSTVd:ΔP-82 controlled by the CoYMV promoter accumulated the second highest amount of 21-nt PSTVd-sRNA, whereas hpPSTVd:Puppo-54 and hpPSTVd:CCRuppo-114, which are both controlled by the CaMV-35S promoter, accumulated the third highest amounts of PSTVd-sRNA of 24-nt and 21-nt, respectively (arrows in Fig. 4a). The accumulations of hpPSTVd:CCRuppo-12, hpPSTVd:CCRuppo-131 and hpPSTVd:CCRdopo-81 were almost at the same levels as the third highest group (data not shown). Other lines accumulated only low levels of PSTVd-sRNA which were hardly visible with approximately 5–10 μg of LMW-RNA for RNA gel blot hybridisation analysis (i.e. these transgenic lines expressed PSTVd-sRNA 10-fold less than those induced by the hpPSTVd:257a-24 and -25 lines).

In order to evaluate the effect of PSTVd infection on the production of siRNAs by the transgenic plants, two transgenic lines, a near full length IR construct (the hpPSTVd-∆TLE transgenic line) that exhibited a low level of PSTVd-sRNA accumulation before inoculation and an hpPSTVd-275a line which showed the highest level of PSTVd-sRNA accumulation, were selected. At 4-weeks post inoculation (wpi), 5 μg of total RNA was analysed by RNA gel blot assay using DIG-labeled PSTVd cRNA probe. As shown in Fig. 4b, hpPSTVd:ΔTLE did not show the presence of any siRNAs in the pre-inoculated plants, but exhibited siRNAs at 4-wpi, indicating that PSTVd infection accumulates PSTVd-sRNA. Similar results were observed in the hpPSTVd-275a-60 line plants, whereas plants derived from both the hpPSTVd-275a-24 and -25 lines showed conspicuous 21- and 24-nt long PSTVd sRNAs before inoculation that was eventually disappeared at 4-wpi (Fig. 4c). Previously, it has shown that PSTVd infection accumulates PSTVd-sRNAs in *N. benthamiana* plants^25^. Interestingly, all of the PSTVd inoculated plants demonstrated the accumulation of PSTVd-sRNA of 21- to 24-nt, with the majority being 22-nt PSTVd-sRNA.

### Evaluation of resistance against PSTVd infection

To evaluate the resistance level shown by each transgenic line against PSTVd, infection assays were performed at least twice using 10–20 seedlings of T3 generations per transgenic line (Table S1). Similar numbers of seedlings were also transformed with an empty vector (EV) and were used as controls. The same amount/concentration of inoculum was used for all of the infection assays: each individual plant was mechanically inoculated with 10 μl of inoculum containing approximately 1 μg of LMW-RNA which contained approximately 100 pg of native PSTVd RNA. Three leaf discs (1-cm in diameter) were collected from the upper most expanded leaf of individual plants at 3-, 4-, and 5-wpi and were used for LWM-RNA extraction. The accumulation of PSTVd RNA in each transgenic plant was verified by subjecting the LMW-RNA to RNA gel blot assays using DIG-labeled PSTVd cRNA as probe. The results showed that although *N. benthamiana* did not exhibit any visible symptoms caused by infection with the PSTVd-I strain used in this analysis, the transgenic lines hpPSTVd:ΔP-82, hpPSTVd:TL-141, hpPSTVd:TL-85, hpPSTVd:TL-173, hpPSTVd:Puppo-54, hpPSTVd:CCRuppo-114, hpPSTVd:257a-24 and hpPSTVd:257a-25 exhibited increased levels of resistance to PSTVd (Table S1). Although the plants were grown in the same growth chamber in the same artificial light conditions, for unknown reasons the germination and the subsequent growth of the seedlings tended to be slower in winter season (November to February). In addition, the infection rate (i.e. the number of plant infected) tended to be lower and inconsistent, even in the control EV line (Table S1).

**Figure 5:**
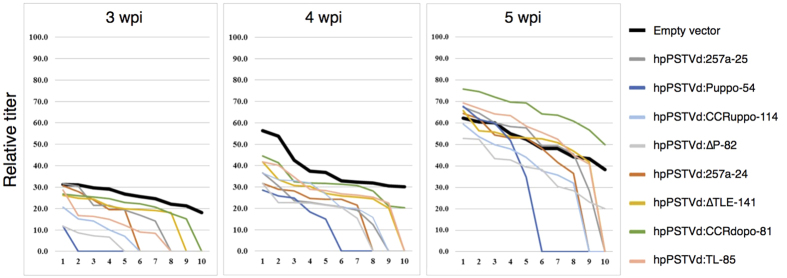
Evaluation of resistance in the selected *N. benthamiana* transgenic lines against PSTVd infection. Ten plants each of the selected eight transgenic *N. benthamiana* lines (hpPSTVd:∆TLE-141, hpPSTVd:∆P-82, hpPSTVd:TL-85, hpPSTVd:Puppo-54, hpPSTVd-CCRuppo-114, hpPSTVd:CCRdopo-81, hpPSTVd:257a-24 and hpPSTVd:257a-25), as well as Empty vector control inoculated with PSTVd, were analysed by gel-blot hybridisation in order to detect the number of plants infected and to determine the relative accumulation levels of PSTVd in each plant at the intervals of 3-, 4- and 5-wpi. The vertical axis indicates the relative PSTVd accumulation levels in the infected plants using a PSTVd-positive control as 100. The horizontal axis indicates plants number 1–10 of each line, from the one showing the highest PSTVd accumulation level to that with the lowest. Those with accumulation level 0 indicate the plants in which PSTVd accumulation level was lower than the current detection limit. The thick black line indicate the Empty vector control. The lines hpPSTVd:Puppo-54, hpPSTVd:∆P-82, hpPSTVd:257a-24, hpPSTVd:257a-25 and hpPSTVd:CCRuppo-114 showed relatively strong resistance. The PSTVd accumulation level was suppressed until 4-wpi in these lines.

For the reasons described above, based on the level of PSTVd-sRNA expression, eight transgenic lines (hpPSTVd:ΔP-82, hpPSTVd:TL-85, hpPSTVd:Puppo-54, hpPSTVd:Puppo-141, hpPSTVd:257a-24, hpPSTVd:257a-25, hpPSTVd:CCRuppo-114 and hpPSTVd:CCRdopo-81) were selected for re-evaluation. All of these plants, along with the EV lines, were grown in the spring and summer (May to June) under the same growth conditions and were challenged with PSTVd as before. The data obtained from two independent infection assays are summarised in Table 2. As shown, five transgenic lines (hpPSTVd:ΔP-82, hpPSTVd:Puppo-54, hPSTVd:Puppo-141, hpPSTVd:257a-24 and hpPSTVd:257a-25) exhibited reduced PSTVd accumulation, indicating increased levels of resistance to PSTVd infection. Furthermore, the amounts of PSTVd RNA accumulated in these plants were analysed by quantifying the intensity of the hybridisation signal using the Quantity One (version 4.6.2) software package. The data was normalised to the amount of RNA loaded on to the gel, and was then compared to that obtained from the EV lines. As shown in Fig. 5, PSTVd titres in the five transgenic lines described above were lower than the EV line up to at least 4-wpi. Furthermore, reduction in the PSTVd titre was more apparent than that observed with the other transgenic lines.

### Effect of the promoter on the production of PSTVd-sRNA and on the level of viroid resistance

**Figure 6:**
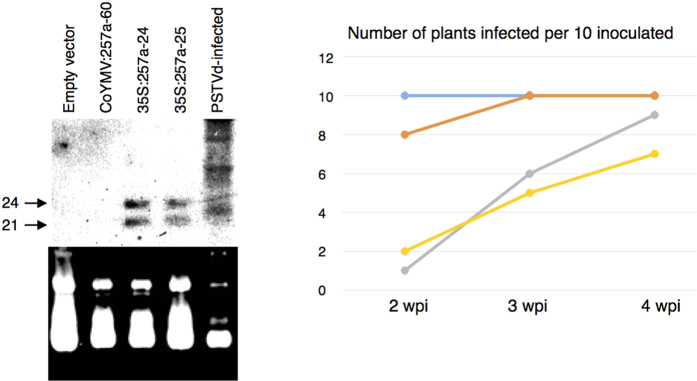
Effect of the promoter on the expression of PSTVd-sRNA and on the resistance against PSTVd in the T3 generation. **(a)** Three *N. benthamiana* transgenic lines expressing hpPSTVd:257a under the control of either the CaMV-35S (hpPSTVd:257a-24 and -25) or the CoYMV (hpPSTVd:257a-60) promoters were analysed for the expression of PSTVd-sRNA before inoculation by gel-blot assay. The upper panel indicates gel-blot hybridisation analysis of PSTVd-sRNA. The arrows indicate PSTVd-sRNA of 24- (upper) and 21- (lower) nucleotides. The lower panel indicates the image of 12% PAGE containing 8M urea stained with ethidium bromide. The arrow indicates t-RNA. The lines under the control of the 35S-promoter accumulated abundant PSTVd-sRNA with the sizes of 24- and 25-nucleotides. **(b)** Ten plants each of the transgenic *N. benthamiana* lines (hpPSTVd:257a-24, -25, and hpPSTVd:257a-60) were challenge-inoculated with PSTVd and the infection analysed at 2-, 3- and 4-wpi. The numbers of plants infected were plotted. The lines under the control of the 35S-promoter expressing abundant PSTVd-sRNA showed resistance to PSTVd infection. In the graph, the blue line indicates empty vector, the orange hpPSTVd:257a-60 line, the grey hpPSTVd:257a-24 and the yellow line hpPSTVd:257a-25, respectively.

In order to compare the effects of two different promoters (CaMV-35S and CoYMV) on both the PSTVd-sRNA production and the level of resistance against viroid infection, two transgenic lines (hpPSTVd:257a-24 and -25) which accumulate an abundance of PSTVd-sRNA under the control of the CaMV-35S promoter and one transgenic line (hpPSTVd:257a-60) which accumulated only low levels of PSTVd-sRNA under the control of the CoYMV promoter, were selected for analysis. In order to evaluate the level of PSTVd-sRNA accumulation, LMW-RNAs extracted from the young expanded leaves of T3 seedlings was subjected to RNA gel blot hybridisation using a DIG-labelled PSTVd cRNA probe. As presented in Fig. 6a, the transgenic lines with the CaMV-35S promoter accumulated more PSTVd-sRNA. In order to verify the correlation between PSTVd-sRNA accumulation and the potential to inhibit PSTVd infection, 10 plants from each of these transgenic lines were challenged by PSTVd infection and subjected to RNA gel blot analysis. As shown in Fig. 6b, the hpPSTVd:257a-24 and -25 lines showed high degrees of resistance towards PSTVd infection at 3-wpi, whereas hpPSTVd:257a-60 failed to show any resistance. In other words, the transgenic lines under the control of the CaMV-35S promoter produced more PSTVd-sRNA and showed greater levels of resistance to PSTVd infection. In contrast, the transgenic lines under the control of the CoYMV promoter accumulated less PSTVd-sRNA and failed to inhibit PSTVd infection.

### Effect of a shorter hpRNA construct stem region on PSTVd infection

To evaluate the level of PSTVd-sRNA production and the amount of resistance to the PSTVd infection by the transgenic plants possessing the IR constructs of shorter than 50-nts, six *N. benthamiana* lines were selected for analysis: two transgenic hpPSTVd:Puppo lines (54 and 141); two hpPSTVd:257a lines (24 and 25), hpPSTVd:CCRdopo-81 and hpPSTVd:CCRuppo-114 expressed hpPSTVd with stem regions of 26-, 40-, 46- and 49-nt in length, respectively. As described above, the *N. benthamiana* lines hpPSTVd:257a-24 and -25, as well as the hpPSTVd:CCRuppo-114 line, produced an abundance of PSTVd-sRNA and exhibited increased levels of resistance to PSTVd infection. This indicates that IR constructs with stem regions as short as 26-nt are capable of inducing siRNA production and therefore offer increased levels of resistance to PSTVd infection.

### Transgenic lines are unable to cross protect closely related viroids

**Figure 7:**
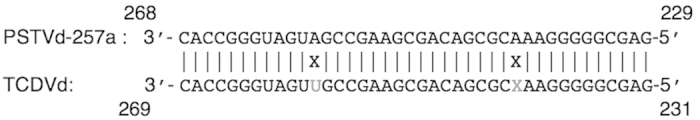
Sequence identity between the hpPSTVd:257a sequence and the corresponding region of TCDVd. TCDVd showed two mismatches (grey in color) in the region of hpPSTVd:257a. Since the sequence was taken from the PSTVd antigenomic sequence, the sequence was placed in the reverse direction (3′–>5′). The numerical numbers on both sides of the sequence indicate the corresponding nucleotides in the genomic sequences of PSTVd and TCDVd.

It was anticipated that the expression of hpRNA derived from highly conserved regions of the PSTVd genome can induce RNA silencing targeting the related *Pospiviroidae* species. Therefore, the two transgenic lines derived from hpPSTVd:257a (24 and 25) were selected because they produce an abundance of vd-sRNA targeting the lower strand of CCR, a region which is highly conserved in the genus *Pospiviroid*. In this case, the small RNAs produced in the transgenic plant were capable of targeting the minus strand of the viroid (replicative intermediate), thus blocking viroid replication. Ten plants each from the two hpPSTVd:257a lines 24 and 25 were challenged by inoculation with *Tomato chlorotic dwarf viroid* (TCDVd), the closest relative of PSTVd (they share 38 of 40 nucleotides in the target region; Fig. 7), and were assayed for the infection using the gel blot hybridisation assay as described for PSTVd infection. All of the plants were similarly infected from 3 wpi, and neither of the transgenic lines exhibited an inhibitory effect on TCDVd infection (Table S2), clearly indicating the transgenic lines developed here are not resistant against closely related viroids.

## Discussion

RNA silencing, a host defence mechanism against invading nucleic acids such as transposons, viruses and transgenes, is triggered by dsRNAs as well as by highly structured single-stranded RNAs (ssRNA) that are processed by Dicer-like ribonucleases into small interfering RNAs (siRNAs)^26^. All known viroid species are composed of a highly base-paired circular ssRNA genome and replicate through a rolling circle mechanism by forming dsRNA replicative intermediates, thus they serve as both potential inducers and targets of RNA silencing^1^,^27^. In fact, dsRNA derived from PSTVd has been shown to inhibit PSTVd infection, probably by RNAi in a sequence-specific manner^14^. Previously, the advantages of creating viroid-resistant plants by a transgenic strategy based on RNA silencing has been reported^15^. However, it was also observed that these same lines of transgenic tomato plants expressing hpRNA derived from a truncated version of PSTVd demonstrated abnormal stunting and leaf curling, similar to that observed in PSTVd infection^18^,^28^. Hence, a total 21 lines of seven different transgene constructs of *N. benthamiana* (hpPSTVd:ΔTLE, hpPSTVd:ΔP, hpPSTVd:TL, hpPSTVd:Puppo, hpPSTVd:CCRuppo, hpPSTVd:CCRdopo and hpPSTVd:257a) expressing various of hpPSTVd sequences under the control of either the CaMV-35S or the CoYMV promoter were created. None of these transgenic lines exhibited any abnormal phenotypes in the T3 generations. In order to eliminate the possibility of the production of these PSTVd-sRNA by latent infection, each transgenic plant was analysed by RT-PCR assay using *Pospiviroid* generic primers^29^. Analysis of sRNA accumulation at 4-wpi revealed that PSTVd infection is associated with sRNA production, which is in agreement with previous data where it was shown that viroid infection accumulates sRNA of 21- to 24-nt, of which the 22-nt species is the most abundant^11^,^20^.

To demonstrate the RNAi mediated resistance offered by the transgenic plants developed here, total RNA extracted from PSTVd infected plants were assayed by RNA gel blot. Though none of the plants were completely resistant, five lines displayed increased levels of resistance to PSTVd infection as is shown by the reduced viroid titres that were observed. These results suggested that hpRNAs consisting of partial PSTVd sequences as short as 26- (hpPSTVd:Puppo) or 40-nt (hpPSTVd:257a) could trigger the RNA silencing of PSTVd without causing adverse effects to the normal growth of the plants when they were expressed in *N. benthamiana*. Recently, the transgenic expression of artificial microRNA (amiRNA) consisting of a 21-nt sequence derived from the upper portion of the P domain of the PSTVd-RG1 strain (nucleotides 46–66) in both *N. benthamiana* and *N. tabaccum* resulted in abnormal phenotypes that were induced by the silencing of the soluble inorganic pyrophosphatase gene expression^19^, suggesting that the sRNA derived from the upper portion of the PSTVd P domain has the potential to disrupt internal host’s gene expression. Although the transgenic line hpPSTVd:Puppo is also derived from the same region of PSTVd, and it exhibited the potential to express PSTVd-sRNAs corresponding to the upper portion of the P domain, it did not cause any abnormal phenotypes in the host plant. Alignment of PSTVd-RG1 with PSTVd-I revealed that, the first two nucleotides (corresponding to nucleotides 46 and 47 of the PSTVd-RG1 genome) in the putative PSTVd-derived amiRNA were different from PSTVd-I (Figure S2), a fact which may be attributed to the difference in the host’s phenotype observed between the two experiments. Hence, no clear conclusion could be drawn on whether or not any possible side effects on the normal gene expression of the host plant can be successfully controlled by using a short PSTVd-related hpRNA strategy, as the PSTVd isolates used in this analysis were symptom-free under the experimental conditions used. Consequently, further analyses are now underway using a tomato cultivar symptomatic of PSTVd infection.

Members of the *Pospiviroidae* family, which replicate via an asymmetric rolling circle mechanism, produce fewer antigenomic strands than genomic strands since these antigenomic strands are only replicative intermediates^30^. Previous high-throughput PSTVd-sRNA sequencing data identified a few hotspot regions in the PSTVd antigenomic strands that produced more PSTVd-sRNAs than did other regions, indicating that these regions are more susceptible to host RNA silencing.^2^,^11^,^20^,^21^. In other words, these regions of PSTVd are less protected against RNA silencing. Theoretically, viroid infection can be efficiently blocked by targeting such low numbered, susceptible regions of the viroid in transgenic plants. As expected, the transgenic *N. benthamiana* lines hpPSTVd:257a-24 and -25, which are transformed with an IR construct that produces an hpRNA with a 40-nt dsRNA stem designed from one of the major hotspot regions of PSTVd-sRNA that is located in the lower CCR–V domain of the antigenomic strand, expressed high levels of PSTVd-sRNAs with two characteristic sizes (21- and 24-nt), and exhibited fairly good resistance to PSTVd infection/accumulation. It should be noted that the final infection rate eventually reached approximately the same level as that of the EV control in most of the transgenic lines (Table 2). Briefly, all of the plants derived from the transgenic lines hpPSTVd-Puppo-141, hpPSTVd:ΔP-82, hpPSTVd:TL-85 and hpPSTVd:CCRdopo-81, and more than 80% of those from hpPSTVd:CCRuppo-114, hpPSTVd-257a:24 and hpPSTVd:257a-25 were infected by 5-wpi. At this point, it is worth considering the fact that mature viroids are known to resist RNA silencing and continue to replicate during RNA silencing of the viroid^8^,^31^. The same mechanism may be underlying these observations; thus, it is essential to clarify the molecular mechanisms of viroid resistance and their escape from RNA silencing.

TCDVd, a member of *Pospiviroidae* family, is the closest relative of PSTVd and shares as much as 90% sequence homology with PSTVd. Hence, two lines of hpPSTVd:257a were infected with TCDVd. Neither exhibited any resistance. Though the biology and host response for TCDVd infection is different from that of PSTVd infection, for RNAi mediated resistance, a near perfect homology is required between the target and the RNA induced silencing complex (RISC) sRNA. The presence of mismatches in the RNA/RNA duplex eventually destabilises the binding of the target to the RISC, thus affecting the overall RNA silencing^32^. Since two mismatches were found in the potential target sequence of TCDVd (Fig. 7), the inability to resist TCDVd infection can be attributed to this difference in the nucleotide sequences.

The expression levels of PSTVd-sRNA greatly differed depending on the transgenic lines selected. Furthermore, even with the same IR construct, the levels of PSTVd-sRNA accumulation also greatly differed among the promoters used. More specifically, the hpPSTVd:257a lines (24 and 25) under the control of the CaMV-35S promoter expressed the highest levels of PSTVd-sRNA and showed greater resistance to PSTVd infection, certainly when compared to the lines (hpPSTVd:257a-60) that controlled by the CoYMV promoter, suggesting that PSTVd-sRNA expression is positively correlated to the extent of the resistance to PSTVd infection. Conversely, hpPSTVd:∆P-82 under the control of CoYMV promoter offered resistance to PSTVd infection similar to that of the hpPSTVd:257a lines (24 and 25) even though it produced less PSTVd-sRNA than do the hpPSTVd:257a lines (24 and 25). Since, hpPSTVd:∆P-82 contains a longer IR, it is capable of producing diverse PSTVd-sRNA which in turn targets different regions of the genomic PSTVd. The hpPSTVd:257a lines contain 40-nt long IR, and are capable of targeting only the lower portion of the CCR and V domains of the antigenomic strand of PSTVd. In other words, siRNA derived from hpPSTVd:∆P-82 targets both infectious and replicative PSTVd RNA, while hpPSTVd:257a lines derived siRNA binds only replicating PSTVd RNA.

Despite all efforts to equate the constructs, the transgenic line under the control of the CaMV-35S promoter was not obtained, at least not in the case of hpPSTVd:ΔTLE which expresses nearly full-length hpPSTVd. Since the CaMV-35S promoter has the potential to express higher hpPSTVd-RNA levels, the possibility cannot be excluded that some of the constructs such as the nearly full-length hpPSTVd might have caused adverse effects on the transformation and regeneration process through RNA silencing^2^,^18^ and that only weakly to moderately expressed lines can be obtained. In this respect, it is also interesting to note that the highest expression levels of PSTVd-sRNA were obtained from the two IR constructs (hpPSTVd:257a-24, -25 and hpPSTVd: ΔP-82), both of which lack the P domain that is responsible for regulating viroid pathogenicity^33^,^34^,^35^. These IR constructs can be candidate transgenes with which to create transgenic plants that inhibit viroid replication, but do not cause adverse effect on the host’s metabolism. However, even though these two IR constructs did not cause any adverse effects on the phenotype of *N. benthamiana,* and successfully expressed higher levels of PSTVd-sRNA, the possibility cannot be excluded that the same constructs expressed in more PSTVd-sensitive hosts such as tomato can induce somewhat adverse effects on the host’s metabolism. In order to clarify this point, experiments using transgenic tomato lines expressing similar IR constructs are now underway.

In conclusion, the results presented here show that it is possible to confer increased resistance to viroids by transforming plants with IR constructs of 26–49 nts in length designed from the partial sequences of both viroid genomic and antigenomic strands. Further analysis will be essential in order to select sequence(s) that have the potential to confer stronger resistance with the appropriate expression levels. This novel technique will definitely improve the strategies available in the RNAi-mediated resistance against various species of viroids, as well as viruses, in a variety of host plants.

## Methods

### PSTVd-sRNA profiling

Using the high-throughput sequence data of tomato plants (*S. lycopersicum*, cv. Rutgers) infected with PSTVd-I (GenBank Acc. No. M16826) and PSTVd-Dahlia (PSTVd-Dah, GenBank Acc. No. AB623143) obtained previously, PSTVd-sRNA hotspots regions were identified on both the PSTVd genomic and antigenomic strands. First, the adapter sequences were removed from the ends of the resulting raw short-read data based on the presence of an exact nucleotide match with the respective adapters, and identical short reads were grouped according to read size (15–29-nt). In this way, trimmed short read data was converted to a non-redundant “short-read-sequence occurrence” format. These non-redundant data were then mapped to either the genomic or the antigenomic strand of PSTVd using hssmap, a specially-written C language program that processes data from circular molecules by adding an appropriate extension to the 3′ terminus of a linear reference sequence as explained before^36^. Based on the profiling, hotspots were determined by visual scanning followed by counting the number of PSTVd-sRNAs located in the particular regions of interest.

### Plasmid construction

A total of seven regions of PSTVd-I were selected for IR constructs on the basis of either of structural/functional domains on the PSTVd-sRNA hotspot data obtained above (Fig. 2). Details of the IR constructs are shown in Figure S3. Briefly, the stretches of ∆TLE, TL, Puppo, CCRuppo and CCRdopo DNA were amplified by PCR using appropriate primer pairs (Table S3), while the DNA construct of ∆P was created by synthetic DNA (Takara Bio Inc., Japan). All of the DNA stretches were sub-cloned in the *Eco*RV site of pBluescript II SK(+) plasmid (Stratagene, La Jolla, CA, USA). The CAT1 intron was amplified by PCR using primers intFw and intRv and was integrated into the *Aat*II/*Hind*III sites of the plasmids containing the ∆TLE, ∆P and TL DNA stretches, as described previously^22^. In order to obtain hairpin constructs in head-to-head orientation, a PCR amplified product of the previously ligated PSTVd DNA sequence was then integrated into the Hind*III*/*Kpn*I sites of the pBluescript II SK(+) plasmid. This final plasmid possesses an insert consisting of a pair of PSTVd sequences in head-to-head orientation that are separated by an intron or a spacer region (*Aat*II restriction endonuclease site). In the case of hpPSTVd:257a, the custom synthesised head-to-head DNA stretch was ligated to pBluescript II SK(+) plasmid at the *Bam*HI/*Hind*III restriction endonuclease sites. The whole construct was digested with *Bgl*II/*Kpn*I and then ligated to pIG121 containing either CoYMV-nos ter or CaMV-35S-nos ter and which was previously digested with the same 2 restriction enzymes (Figure S3a and S3b). In case of hpPSTVd:257a, the custom synthesised head-to-head DNA stretch was ligated to pBluescript II SK(+) plasmid at the *Bam*HI/*Hind*III restriction endonuclease sites. This was later integrated into pIG121 having either CoYMV-nos ter or CaMV-35S-nos ter, in between the *Bam*HI/*Hind*III restriction endonuclease sites (Figure S3c). Empty vector was constructed according to Kasai *et al.*^37^ and was used as a control. The DNA sequences of the plasmids were confirmed using an ABI 3500 GA sequencer (Applied Biosystems Inc.).

### Agrobacterium-mediated transient expression assay

All of the pIG121 binary vectors containing IR constructs were transformed into *Agrobacterium tumefaciens* strain EHA105. Agrobacterium cells containing the IR-expressing binary vector were inoculated in an induction solution and incubated overnight at 28 °C as described previously^38^. Bacterial cells were collected by centrifugation at 3000 × g for 15 min at 25 °C, and were then resuspended in infiltration buffer (10 mM MgCl_2_, 10 mM MES, and 200 mM acetosyringone) and adjusted to an optical density at 600 nm of 1.0. They were maintained at room temperature for 4 h before agroinfiltration in to *N. benthamiana* leaves. At 3-dpi, total RNA was extracted from the infiltrated leaf areas and was analysed for PSTVd-sRNA production by RNA gel blot.

### Plant transformation, selection of transgenic plants and growth conditions

*N. benthamiana* plants were transformed by the leaf disc method using *Agrobacterium tumefaciens*^39^. The single functional T-DNA insert and the presence of the IR-RNA structure were identified by a 3:1 segregation for kanamycin resistance in the seeds (T1 generation) obtained from the self-pollinated primary transformants. The regenerated plants were transferred to pots and maintained at 28 °C. T0 generation plants were screened by PCR for the presence of transgene constructs, and positive lines were maintained for the production of the homozygous T2 generation. Homozygosity of the integrated gene in the 2^nd^ generation of inbred lines was confirmed by the absence of antibiotic sensitivity and by PCR amplification of the transgenic constructs as described earlier^22^. These transgenic lines were used for the T3 generation. Two weeks after sowing seeds, transgenic seedlings were transferred to rockwool (Nitto Boseki Co., Tokyo, Japan) in a standard nutrient solution (Ohtsuka House, Ohtsuka Chemical Co., Osaka, Japan) and grown in a plant growth room at 25 °C under 16 h light/8 h dark conditions (Figure S4).

### Viroid infection assays in transgenic *N. benthamiana* plants

Inoculum was prepared at a concentration of 100 ng/μl of LWM-RNA (containing ca. 100 pg/μl of PSTVd) extracted from PSTVd-I infected tomato plants (*S. lycopersicum*, cv. Rutgers) in 10 μl of 50 mM sodium phosphate buffer (pH 7.5) containing 1 mg/ml bentonite. Two leaves of the 3 week old T3 generation *N. benthamiana* plants (7–8 leaf stage) were dusted with carborundum (600 mesh), and then 5 μl of inoculum (10 μl/plant) was placed on the leaf and gently rubbed against it 20 times using a sterile glass rod. The inoculated leaves were then rinsed with distilled water and maintained in the greenhouse as above. Ten seedlings per transgenic line were used for one infection assay, and the assay was repeated at least twice except for the TCDVd infection assay.

### RNA extraction and gel blot assay

For the verification of PSTVd-sRNA production in the transgenic lines, leaves (approx. 1 g) were collected prior to viroid inoculation. The samples were ground into fine powders with a sterilized motor and pestle using liquid nitrogen, and then total RNA was extracted by 2 × CTAB buffer as described previously^36^. LMW-RNA containing viroid and viroid specific sRNAs were then enriched by 2M LiCl fractionation followed by phenol:chloroform (1:1) extraction. The RNA was precipitated by adding 2.5 volumes of absolute alcohol and was quantified by spectrophotometer at 260 nm. Samples (5 μg/ 20 μl) of enriched RNA were mixed with 0.9 volumes 50% (w/v) urea solution and 0.1 volumes of loading dye solution [0.02% (w/v) bromophenol blue, 0.02% (w/v) xylene cyanol, 50% (v/v) glycerol] and then were heat denatured at 68 °C for 15 min and the small RNAs separated by polyacrylamide 12% (w/v) polyacrylamide gel electrophoresis (PAGE; acrylamide:bisacrylamide = 19:1) in buffer containing 1 × Tris–borate-EDTA (TBE) buffer and 8 M urea. The RNAs were then transferred to nylon membrane (Biodyne Plus) and then hybridised with a DIG-PSTVd cRNA probe as described previously^40^. The presence of 21- and 24-nt long PSTVd-sRNAs was verified by referring to the RNA size maker.

For the detection of PSTVd in challenged transgenic *N. benthamiana* plants leaf discs were collected from the upper most of fully expanded leaves of each plant at 3-, 4- and 5-wpi and the total RNA extracted and fractionated by 2 M LiCl as described above. An RNA sample of 125 ng was dissolved in 5 μl of loading buffer containing 10% (v/v) glycerol and 0.01% each of bromo-phenol blue and xylene cyanol FF and heat denaturation at 68 °C for 15 min. The samples were then separated by electrophoresis in 1.5% agarose-formamide gels containing 1X MOPS (3-[N-morpholino] propanesulfonic acid) buffer. The RNAs were then transferred to a nylon membrane (Biodyne Plus, Pall Corp.) and hybridised with digoxigenin-labeled riboprobe in order to detect PSTVd at 55 °C for 16 h. The LWM-RNA used for the inoculation was used as positive control. Northern hybridisation signals were visualised using a Chemidoc-XRS (Bio- Rad) imaging system (1 to 2 h exposure), and were quantified using the Quantity One (version 4.6.2) software package.

## Additional Information

**How to cite this article**: Adkar-Purushothama, C. R. *et al.* RNAi mediated inhibition of viroid infection in transgenic plants expressing viroid-specific small RNAs derived from various functional domains. *Sci. Rep.* **5**, 17949; doi: 10.1038/srep17949 (2015).

## Supplementary Material

Supplementary Information

## Figures and Tables

**Table 1:** List of transgenes, corresponding functional domains and PSTVd-sRNA hotspots and line number of the transgenic plants.

Name of Transgene	Functional domainof viroid	Hotspot of PSTVd-sRNAto express	Length of sequencein nt*	Promoter toexpress transgene	Line No. ofTransgenic plant
hpPSTVd:ΔTLE	Lacks terminal end of upper and lower TL	Almost the same to PSTVd	340	CoYMV	24
					32
					141
hpPSTVd:TL	TL	P1 +M1	91	CaMV-35S	141
				CoYMV	85
					173
hpPSTVd:ΔP	Lacks upper and lower P	Lacks P2	281	CaMV-35S	71b
				CoYMV	82
hpPSTVd:Puppo	Upper P	P2	26	CaMV-35S	54
					122
					141
					161
hpPSTVd:CCRuppo	Upper CCR	P3 + M2	49	CaMV-35S	65
					114
				CoYMV	12
					131
hpPSTVd:CCRdopo	Lower CCR	P4 + M5	46	CaMV-35S	81
hpPSTVd:257a	Portion of lower CCR and V	M5	40	CaMV-35S	24
				CoYMV	25
					22
					60

^*^Length of the PSTVd sequence used to construct the IR.

**Table 2:** PSTVd infection assay performed on the selected transgenic lines of *N. benthamiana* which showed higher levels of resistance in the preliminary experiments.

Transgenic line	3-weeks post inoculatuin*1	4-weeks post inoculatuin*1*	5-weeks post inoculatuin*1*
hpPSTVd:∆TLE-141	7/9*2	9/9	9/9
hpPSTVd:∆P-82	4/10	7/10	10/10
hpPSTVd:TL-85	7/9	9/9	9/9
hpPSTVd:Puppo-54	1/9	5/9	5/9
hpPSTVd:CCRuppo-114	5/10	8/10	8/10
hpPSTVd:CCRdopo-81	9/10	10/10	10/10
hpPSTVd:257a-24	5/10	6/10	8/10
hpPSTVd:257a-25	7/10	8/10	9/10
Empty vector	10/10	10/10	10/10

^*1^Number of plants infected/inoculated.

^*2^Ten plants per transgenic line were inoculated. However, since some of the plants grew poorly for accidental reasons (probably physiological problems) in some lines, they were eliminated from the analysis.
