# Supplementary material for: RNAi mediated inhibition of viroid infection in transgenic plants expressing viroid-specific small RNAs derived from various functional domains
Source: Sci Rep. 2015 Dec 14;5:17949. doi: 10.1038/srep17949 (PMC4677296; doi:10.1038/srep17949)
Supplement: Supplementary Information [file srep17949-s1.pdf]

Supplemental Information

Table S1 Preliminary evaluation of hpPSTVd transgenic lines by PSTVd infection assay.

Transgene	Trsngenic line No.	3-wpi	4-wpi	5-wpi
	24	8/10[a]	9/10	NT
hpPSTVd-ΔTLE	32	8/10	8/10	NT
	141	7/10	7/10	NT
Empty control		8/10	10/10	NT
hpPSTVd-ΔP	71b	0/20	3/20	6/20
	82	0/20	1/20	3/20
Empty control		0/20	2/20	8/20
hpPSTVd-TL	144	0/20	17/20	NT
	85	0/20	10/20	NT
	173	1/15	11/15	NT
Empty control		7/20	14/20	NT
hpPSTVd-P-uppo	54	4/20	15/20	16/20
	122	14/20	19/20	19/20
	141	11/20	12/20	17/20
	161	14/20	18/20	20/20
Empty control		17/20	18/20	19/20
hpPSTVd-CCR-uppo	65	18/20	18/20	19/20
	114	13/20	18/20	19/20
	12	13/20	17/20	20/20
	131	14/20	19/20	19/20
Empty control		17/20	18/20	19/20
hpPSTVd-CCR-dopo	81	14/20	14/20	15/20
Empty control		17/20	18/20	19/20
hpPSTVd-257a	24	3/10	9/10	10/10
	25	1/10	5/10	7/10
	22	NT[b]	NT	NT
	60	7/10	9/10	10/10
Empty control		10/10	10/10	10/10

[a] Number of plants infected/inoculated

[b] Not tested

Table S2 Challenge inoculation assay of transgenic *N. benthamiana* lines expressing hpPSTVd-257a by TCDVd for analyzing cross protection against related viroid.

Transgene	Transgenic line (promoter)	3-wpi [a]	4-wpi	5-wpi
hpPSTVd:257a	24 (CaMV-35S)	10/10 [b]	10/10	10/10
	25 (CaMV-35S)	10/10	10/10	10/10
Empty vector	(CaMV-35S)	10/10	10/10	10/10

[a] weeks post inoculation.

[b] Number of plants infected/inoculated

Table S3 List of oligonucleotides used in this study for preparing the IR constructs.

Name	RE site addition	Sequence	Product
intFW [a]	*Aat*II	GCGACGTCCTACAGGGTAAATTCTAGTTTTTC	CAT1 intron
intRV [a]	*Hind*III	GCGACGTCCTACAGGGTAAATTCTAGTTTTTC	
tPSTVd sFW [a]	*Bgl*II	GCAGATCTGGTTCCTGTGGTTCACAC	hpPSTVd:ΔTLE and hpPSTVd:ΔP
tPSTVd sRV [a]	*Aat*II	GCGACGTCACCAACTGCGGTTCCAAG	
tPSTVd aFW [a]	*Hind*III	GCAAGCTTACCAACTGCGGTTCCAAG	
tPSTVd aRV [a]	*Kpn*I	CTGGTACCGGTTCCTGTGGTTCACAC	
TL sFPBg	*Bgl*II	GCAGATCTCTTCGGGGCGAGGGTG	hpPSTVd:ΔTL
TL sRPA	*Aat*II	GCGACGTCCTCAGGAGGTCAGGTGTG	
TL aFPH	*Hind*III	GCAAGCTTCTCAGGAGGTCAGGTGTG	
TL aRPK	*Kpn*I	CTGGTACCCTTCGGGGCGAGGGTG	
P Suppo FPBg	*Bgl*II	TTAGATCTCAGAAAAGAAAAAAGAAGG	hpPSTVd:Puppo
P Suppo RPA	*Aat*II	GCGACGTCCGAGCCGCCTTCTTTTTTC	
P Auppo FPA	*Hind*III	GCGACGTCCGAGCCGCCTTCTTTTTTC	
P Auppo RPK	*Kpn*I	CTGGTACCCAGAAAAGAAAAAAGAAGG	
CCR Suppo FPBg	*Bgl*II	TTAGATCTGAGGAGCGCTTCAGGGATCCCCGGGG	hpPSTVd:CCRuppo
CCR Suppo RPA	*Aat*II	GCGACGTCTTTTGCCAGTTCGCTCCAGGTTTCCCCG	
CCR Auppo FPA	*Hind*III	GCGACGTCTTTTGCCAGTTCGCTCCAGGTTTCCCCG	
CCR Auppo RPK	*Kpn*I	CTGGTACCGAGGAGCGCTTCAGGGATCCCCGGGG	
CCR Sdopo FPBg	*Bgl*II	TTAGATCTTGCGCTGTCGCTTCGGCTACTACCCGG	hpPSTVd:CCRdopo
CCR Sdopo RPA	*Aat*II	GCGACGTCGGAGCTTCAGTTGTTTCCACCGGG	
CCR Adopo FPA	*Hind*III	GCGACGTCGGAGCTTCAGTTGTTTCCACCGGG	
CCR Adopo RPK	*Kpn*I	CTGGTACCTGCGCTGTCGCTTCGGCTACTACCCGG	

siR257a	*Bam*HI, *Kpn*I	TTGGATCCCACCGGGTAGTAGCCGAA GCGACAGCGCAAAGGGGGCGAGTTTC CTCGCCCCCTTTGCGCTGTCGCTTCGG CAACTACCCGGTGGGTACCTT	hpPSTVd:257a

[a] Kasai et al., [1]

Underlined nucleotides denote restriction endonuclease site.

Small lettered nucleotides denote the linker sequence.

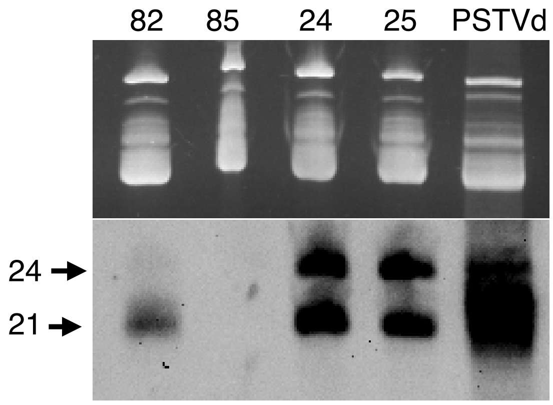

Figure S1 Gel-blot hybridization analysis of PSTVd-sRNA. The arrows indicate the PSTVd-sRNA of 21 – 24-nts. The amount of PSTVd-sRNA produced by the various transgenic lines was quantified. The relative expression levels of PSTVd-sRNA were variable depending on the lines. *Lane 82* hpPSTVd:ΔP-82, *lane 85* hpPSTVd:TL-85, *lane 24* hpPSTVd:257a-24, *lane 25* hpPSTVd:257a-25 and *lane PSTVd* PSTVd inoculated plant. The numbers below each lane represent the μg of RNA charged per lane.

Figure S1 (Sano)

```
                35                                        70
PSTVd-I:         5′- UGACCUCCUGAGCAGAAAAGAAAAAAGAAGGCGGCU -3′
                     |||||||||||xx|||||||||||||||||||||||
PSTVd-RG1:       5′- UGACCUCCUGACAAGAAAAGAAAAAAAGAAGGCGGCU -3′
                35                                         70
```

Figure S2 Sequence identity between the PSTVd-I used to construct the IR expressing hpPSTVd:Puppo and the corresponding region of the PSTVd-RG1 isolate. The underlined nucleotides represent the amiR46 sequence[1]. The two mismatches are shown by X in the region. The numerical numbers on both sides of the sequence indicate the corresponding nucleotides in genomic sequences of PSTVd-I and PSTVd-RG1.

Figure S2 (Sano)

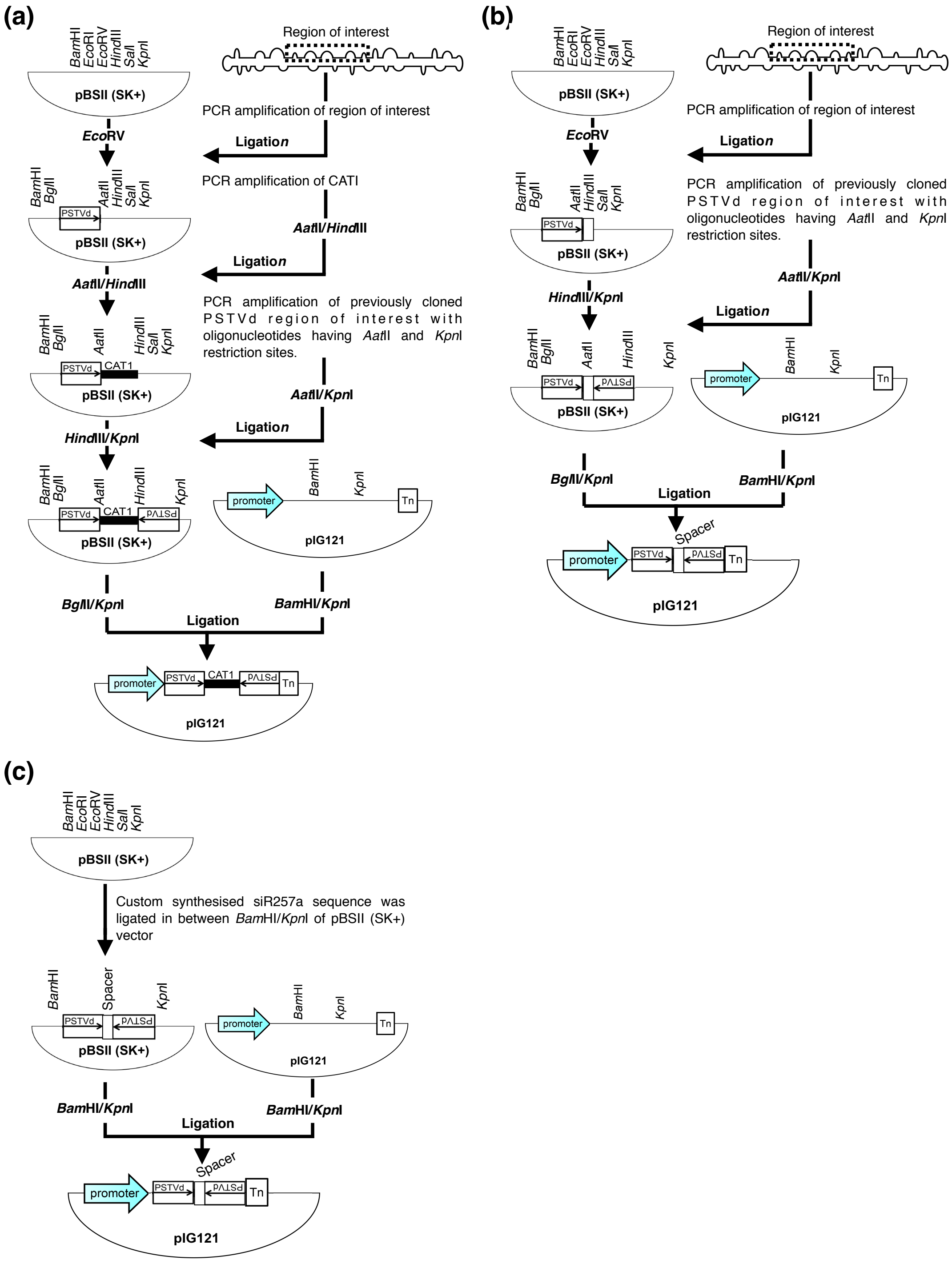

Figure S3 Diagrammatic representation of steps involved in the preparation of the hairpin constructs derived from the various regions of PSTVd. The arrows inside the box indicate the direction of PSTVd sequence used. The IR sequence of the PSTVd is separated by (a) an intron for hpPSTVd:ΔTLE, hpPSTVd:ΔP and hpPSTVd:TL; while a small sequence was used as spacer for (b) hpPSTVd:Puppo, hpPSTVd:CCRuppo, hpPSTVd:CCRdopo; and, (c) si257a, respectively. The whole construct was inserted between the promoter (either CaMV-35S or CoMYV) and the Tn (nopaline synthase terminator) sequence of the vector pIG121.

Figure S3 (Sano)

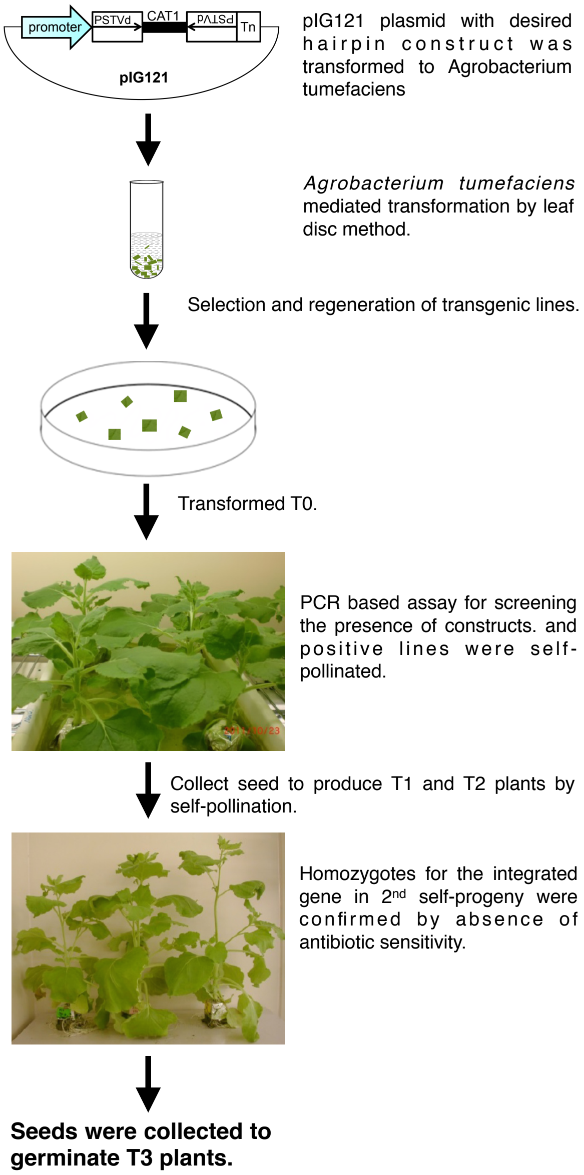

Figure S4 The steps involved in the production of transgenic lines and in the subsequent generation of the T3 lines are shown schematically.

Figure S4 (Sano)

Supplemental information References

1. Kasai, A., Sano, T. & Harada, T. Scion on a Stock Producing siRNAs of Potato Spindle Tuber Viroid (PSTVd) Attenuates Accumulation of the Viroid. *PLoS One* **8,** e57736 (2013).
